# Association between parents’ concerns about eating and sleeping problems and social-emotional development in Chinese children aged 3 to 6 years

**DOI:** 10.3389/fpubh.2023.1264219

**Published:** 2023-11-16

**Authors:** Tongxi Hu, Shaoying Liu, Jianying Zhan, Luxin Xu, Yanqing Zhou

**Affiliations:** ^1^Department of Psychology, Zhejiang Sci-Tech University, Hangzhou, China; ^2^Department of Child Health Care, Hangzhou Women’s Hospital, Hangzhou, China; ^3^Hangzhou Qiantang Lingyun Kindergarten, Hangzhou, China; ^4^Hangzhou Qiantang Xinghua Kindergarten, Hangzhou, China

**Keywords:** preschool children, parental concern, eating problem, sleeping problem, social-emotional development

## Abstract

**Background:**

Parents’ parenting beliefs have a major influence on their children’s eating and sleeping problems and emotional socialization. However, the relationship between parent’s concerns about eating or sleeping problems and social-emotional development is unclear.

**Methods:**

We used a convenience sampling method to investigate 997 parents of preschool children aged 3 to 6 in Hangzhou, China, and asked them to complete the “Ages & Stages Questionnaire: Social-Emotional (2nd Edition)” (ASQ: SE-2) and the Survey of Concerns about Children’s Eating and Sleeping Problems. To examine the relationship between children’s social-emotional development and their parents’ concerns about their eating or sleeping problems, binary logistic regression was used.

**Results:**

There were 218 children (21.9%) with a suspected social-emotional development delay, and 273 parents (27.4%) were concerned about their children’s eating or sleeping problems, which mainly focused on ill-balanced eating, bad eating habits, and difficulty falling asleep. The rate of suspected social-emotional development delay in children with the co-occurrence of eating and sleeping problems (37.8%) was significantly higher than those with only eating problems (29.7%), only sleeping problems (24.4%), and those with no eating or sleeping problems (18.8%) (*p* < 0.05). A binary logistic regression analysis showed that parents’ concerns about the co-occurrence of eating and sleeping problems (OR = 2.52, *p* = 0.01) and only eating problems (OR = 1.71, *p* = 0.004) were risk factors for children’s social-emotional development. In addition, boys were more likely than girls to have suspected social-emotional development delay (OR = 1.49, *p* = 0.01).

**Conclusion:**

Children whose parents were concerned about only eating or the co-occurrence of eating and sleeping problems were linked to have a higher risk of suspected social-emotional development delay.

## 1 Introduction

Early childhood is a critical period for the formation of lifelong eating and sleeping behaviors and patterns (1). By the time children are 3 or 4 years old, eating and sleeping is not only deprivation-driven, but also influenced by their responsiveness to environmental cues, particularly caregiver interaction experience (2). Unfortunately, eating and sleeping problems are quite common in young children (3, 4). The eating problems among young children include 7 aspects: ill-balanced eating, food responsiveness, bad eating habits, satiety responsiveness, external factor eating, emotional eating, and initiative eating (4). Similarly, the sleeping problems are usually characterized by difficulties in sleep onset, sleep maintenance and sleep quality despite adequate sleep conditions, such as sleeping onset delay, night waking and bedtime resistance (5). Studies have found that the prevalence of eating problems in young children is estimated to be between 25 and 45% (6), while the prevalence of sleeping problems ranges from 20 to 65% (5). Eating problems in young children are associated with an increased likelihood of sleeping problems (7, 8). It has been established that eating or sleeping problems not only have a negative correlation with the physical growth of young children (9), but also their social-emotional development. A cross-sectional study of 7,179 three-year-old children found that those who had a partial eclipse, such as eating vegetables less frequently, scored higher in social-emotional problems (10). Additionally, a case-control study showed that 3- to 6- year-olds who got inadequate sleep (less than 9 h) and went to bed late (after 23:01) had higher scores for social-emotional problems (11).

Early childhood is a key period for social-emotional development (12) and parenting beliefs and practices play a significant role in this development (13). Parents encounter struggles and hardships while caring for their young children’s eating and sleeping, leading to unfavorable parent-child interactions (14). Parents’ concerns about their young children’s eating and sleeping problems reflect their parenting efficacy and satisfaction related to caring for their children’s eating and sleeping, which are important factors that affect young children’s social-emotional development (15). Existing research has shown that parenting sense of competence (parenting efficacy and satisfaction) is negatively correlated with children’s eating and sleeping problems (1), while positively correlated with children’s social-emotional development (16). However, most studies often measured young children’s eating and sleeping problems through scales or observations, and separately examined the relationship between an only eating or sleeping problems and young children’s social-emotional development separately. Therefore, it is important to explore the relationship between eating and sleeping problems simultaneously and social-emotional development in young children from the perspective of parental concerns, and further understand the relationship between only eating, only sleeping, the co-occurrence of eating and sleeping problems and social-emotional development in young children. The relationship between these factors may reflect the larger family environment in which young children live, which could help inform public health policies aimed at improving child health and well-being.

## 2 Methods

### 2.1 Sampling procedure

This study was conducted in six kindergartens with 1,536 children in Hangzhou, Zhejiang Province, China, using a convenience sampling method. The questionnaire was approved by each principal and distributed to the parents of preschool children who were willing to participate in the survey, with instructions that specified the aim of the survey and ensured privacy protection. The questionnaire was filled out online.^1^ After scanning the QR code, the parents completed the questionnaire and submitted it. A week later, the parents received the feedback report of the questionnaire they filled out via email.

We collected the emails and contact details of 1,002 children aged 3–6 whose parents reported their eating and sleeping problems and social-emotional development. Out of these, 997 valid questionnaires were collected, giving an effective recovery rate of 99.5%. We excluded questionnaires with the repeated submissions and children who were not within the age range of the social-emotional development screening questionnaire.

This study has been approved by the Psychology Ethics Committee of Science at Zhejiang Sci-Tech University. The data collection process complies with the principles and requirements of ethics.

### 2.2 Demographics

This section included questions related to the child’s age, gender, birth order and caregiving mode. Since China implemented the two-child and three-child policies, the percentage of second- and third-child births in the population is about 50% in 2021 (17). Therefore, the birth order of young children was divided into 2 categories: first-born, second-born and above. According to a survey of 24,366 children aged 3–6 in 2021 (18), the caregiving mode of young children was divided into three groups: parents alone, parents and grandparents (or nannies similar in age to the grandparents) raising them together, and grandparents raising them alone. Of these, 49.10% were being taken care of by parents and grandparents.

### 2.3 Social-emotional development

Parents of children filled out the Chinese version of the Ages & Stages Questionnaire: Social Emotional II (ASQ:SE-2) in order to evaluate their children’s social-emotional development. The questionnaire was developed by Professor Jane Squires and revised by Xiaoyan Bian and others in 2017, with good reliability and validity (the reliability Cronbach’s *α* = 0.77, test–retest reliability *r* = 0.77, and the structural validity was 0.84) (19, 20). ASQ:SE-2 has different forms for each age group, with items that cover the age intervals of 36 months (33 months 0 days to 41 months 30 days; 31 items), 48 months (42 months 0 days to 53 months 30 days; 33 items), and 60 months (54 months 0 days to 71 months 30 days; 33 items). Each item was rated using a three-point Likert scale (10 = most of the time, 5 = sometimes, 0 = never or rarely) and an additional score of 5 was added when the item was concerning for parents. If the total score was equal to or higher than the cut-off point, the child is considered to have a “suspected social-emotional development delay” and will be advised to receive further professional evaluation.

The Cronbach’s α of the three questionnaires in this study was 0.84, 0.73, and 0.74, respectively.

### 2.4 Parents’ concerns about children’s eating and sleeping problems

We asked the question “Do you have any concerns about your child’s eating and sleeping behaviors?” to identify the concerns that parents have about their children’s eating and sleeping problem. If the answer was “Yes,” we inquired about the specific eating or sleeping problems that caused them concern. Parental answers were coded as follows, based on classifications from previous behavioral studies on eating and sleeping problems (21–24).

Eating problems were divided into 7 categories, such as: ① Ill-balanced eating: eat fewer vegetables, eat fewer staple foods, eat fewer meat dishes, etc., ② Bad eating habits: eat too quickly or too slowly, eat while watching TV, eat while playing, etc., ③ Satiety responsiveness: easy to get full, not greedy when they see food, have a lack of appetite, and a smaller appetite than ordinary children, ④ Food responsiveness: eat whatever is given to them at any time, have a strong appetite, get hungry easily, and eat more food than ordinary children, ⑤ External factor eating: eat more outside than at home, eat more when there are many sets of tableware or meals, ⑥ Initiative eating: being unable to eat independently, and not bringing food for oneself when eating, etc., ⑦ Craving for junk food: having a strong love for snacks, etc.

Sleeping problems were divided into 9 categories, such as: ① Difficulty falling asleep: sleep time > 30 min, ② Enuresis: bedwetting, ③ Insomnia: sleep time at night <9 h, ④ Inability to sleep normally, ⑤ Reject quilt cover, ⑥ No nap, ⑦ Easy to wake up: such as shallow sleep, ⑧ Parents sleeping together: unable to sleep in separate rooms with parents, and ⑨ Night terrors: such as crying at night.

Two people independently executed the coding work and the Kappa consistency test was used to evaluate the consistency of the classification, yielding a coefficient of 0.906.

### 2.5 Data analysis

This research used descriptive and comparative approaches to explore the factors associated with children’s social-emotional development. Descriptive and frequency statistics were applied to analyze the general variables, while chi-square tests were used to compare the different groups. The variables that showed statistically significant differences in the univariate analysis were then included as independent variables in a binary logistic regression model. The strength of the association was indicated by the Odds Ratio (OR) value and its 95% confidence interval. All statistical tests were two-sided and conducted at a significance level of 0.05. The data analysis was conducted using SPSS Version 22.0.

## 3 Results

This study included 997 Chinese children with 53.2% boys and 46.8% girls, and the mean age was 57.7 months (SD = 8.0 months). Of the participants, 2.8% were 36-month-old (M = 37.9 months, SD = 2.4 months), 27.4% were 48-month-old (M = 49.3 months, SD = 2.9 months), and 69.8% were 60-month-old (M = 61.9 months, SD = 5.1 months). The majority of the children (69.8%) were the first-born child in their families. Parents were the primary caregiver for 39.1%, while parents and others were caregivers for 60.9%.

According to the reports of parents, 27.4% of the children were concerned about their eating or sleeping problems; 19.6% had only eating problems, 4.1% had only sleeping problems, and 3.7% had the co-occurrence of eating and sleeping problems. Table 1 displays frequencies and percentages of children’s eating and sleeping problems. Parents reported a total of 280 and 77 frequencies, respectively, for children’s only eating or only sleeping problems. The most common eating and sleeping problems were ill-balanced eating (36.8%), bad eating habits (26.7%), and difficulty in falling asleep (48.1%). Based on parental report on the ASQ:SE-2, 21.9% (218/997) of children were identified with suspected social-emotional development delay.

**Table 1:** Frequencies and percentages of children’s eating or sleeping problems by parents reported.

**Eating problem**	**Frequency**	**Percentage (%)**	**Sleeping problem**	**Frequency**	**Percentage (%)**
Ill-balanced eating	103	36.79	Difficulty falling asleep	37	48.05
Bad eating habits	73	26.07	Enuresis	15	19.48
Satiety responsiveness	42	15.00	No nap	9	11.69
Initiative eating	42	15.00	Insomnia	6	7.79
Food responsiveness	9	3.21	Parents sleeping together	3	3.90
Craving for junk food	6	2.14	Night terrors	2	2.60
External factor eating	5	1.79	Easy to wake up	2	2.60
			Inability to sleep normally	2	2.60
			Reject quilt cover	1	1.30
Total	280	100.00	Total	77	100.00

Table 2 displayed that gender, caregiving mode, and parents’ concerns about eating or sleeping problem were found to have a significant difference on the rate of suspected social-emotional development delay in children. However, there was no significant difference due to month and birth order.

**Table 2:** Disparities of social-emotional development on participant’s characteristics and parents’ concerns about eating or sleeping problems.

	Social-Emotional development *n* (%)		*χ* ^2^	*p*
	Below cut-off point (*n* = 779)	Above cut-off point (*n* = 218)		
Characteristics				
Gender			5.385	0.020
Boys	399 (75.3)	131 (24.7)		
Girls	380 (81.4)	87 (18.6)		
Age (months)			5.676	0.059
36	20 (71.4)	8 (28.6)		
48	201 (73.6)	72 (26.4)		
60	558 (80.2)	138 (19.8)		
Birth order			0.406	0.524
First-born	540 (77.6)	156 (22.4)		
Second-born and above	239 (79.4)	62 (20.6)		
Caregiving mode			12.504	0.002
Grandparents	58 (75.3)	19 (24.7)		
Parents and others	393 (74.3)	136 (25.7)		
Parents	328 (83.9)	63 (16.1)		
Variable				
Parents’ concerns about eating or sleeping problem			16.785	0.001
None	588 (81.2)	136 (18.8)		
Only eating problem	137 (70.3)	58 (29.7)		
Only sleeping problem	31 (75.6)	10 (24.4)		
Co-occurrence of eating and sleeping problems	23 (62.2)	14 (37.8)		

Binary logistic regression showed that children with only eating problems (OR = 1.71), and those with the co-occurrence eating and sleeping problems (OR = 2.52) had a higher risk of suspected social-emotional development delay than those without any problems. Furthermore, boys had a higher risk of suspected social-emotional development delay than girls (OR = 1.49) (Table 3).

**Table 3:** Logistic regression model of factors associated with suspected social-emotional development delay (*N* = 997).

Variables	β	S−*x*	Wald χ^2^	*p*	OR (95% CI)
Gender					
Girls					Ref.
Boys	0.401	0.159	6.377	0.012	1.494 (1.094 ~ 2.039)
Parents’ concerns about eating or sleeping problem					
None					Ref.
Only eating problem	0.535	0.186	8.286	0.004	1.708 (1.186 ~ 2.460)
Only sleeping problem	0.280	0.380	0.543	0.461	1.323 (0.628 ~ 2.788)
Co-occurrence of eating and sleeping problems	0.924	0.358	6.680	0.010	2.520 (1.250 ~ 5.079)

## 4 Discussion

In this study, we found a significant association between 3- to 6-year-olds’ eating and sleeping problems that parents were concerned and social-emotional development. It gives us an idea of the relationship between young children’s eating and sleeping problems and social-emotional development from the perspective of parenting beliefs. Here, we discuss our findings and give proposals for practice and research.

Results showed that 27.4% parents who were concerned about their children’s eating or sleeping problems. Out of this group, 19.1% reported their children’s only eating problems, 4.1% reported only sleeping problems, and 3.7% reported the co-occurrence of both. However, this is lower than the rates of eating problems (25 to 45%) (6) and sleeping problems (20 to 65%) (5) reported in young children in previous studies. This discrepancy might be due to the fact that this study examines children’s eating and sleeping problems from the perspective of parents’ concerns. Parents’ concerns stem from lower parenting efficacy or lower satisfaction in caring for children with eating and sleeping problems, even though some young children may have eating or sleeping problems, but their parents have no concerned about these. In this study, the parents’ concerns about eating or sleeping problems with higher proportion were ill-balanced eating, bad eating habits and difficulty falling asleep, which is consistent with the findings of previous studies (3, 6).

This study indicated that children whose parents were concerned about only eating or co-occurrence of eating and sleeping problems were at higher risk for suspected social-emotional development delay compared to those whose parents had no such concerns. Specifically, the risk of suspected social-emotional development delay in children whose parents are reported only eating problems is 1.71 times and children who have the co-occurrence of eating and sleeping problems is 2.52 times higher than those who had no such concerns separately. Previous studies have consistently demonstrated that young children with eating or sleeping problems, such as ill-balanced eating and difficulty falling asleep, are more likely to have suspected social-emotional development delay (25). Good eating or sleeping habits, including balanced food intake and regular meal or sleep time, are beneficial to children’s physical and mental health (26). This study did not find a relationship between a only sleep problem and social-emotional development in young children, which is contrary to previous research (11). This may be because the parents in this sample reported lower rates of only sleeping problems and some parents were not concerned about sleeping problems in their children. Parents may experience challenges and frustrations when caring for children with eating or sleeping problems. Existing research has demonstrated that these problems are connected to the caregiver concerns (27). Parents’ concerns reflect their low parenting efficacy and satisfaction in eating and sleeping care, which is a result of their lack of parenting sense of competence. Studies have shown that there is a negative correlation between parenting sense of competence and their children’s social-emotional development (16). Examining the relationship between young children’s eating and sleeping problems and their social-emotional development from the perspective of parental concern is essential for the promotion of physical and mental well-being in young children.

In order to prevent and intervene young children’s social-emotional development, our study suggests that interventions should not only focus on the child’s social-emotional development, but also on parenting sense of competence in caring for their children’s eating and sleeping. Future research should develop successful family-oriented approaches to tackle eating and sleeping problems, which could reduce parental anxiety and enhance the parent-child bond. It is essential for child care professionals and policy makers to recognize the importance of parenting sense of competence in providing for their children’s eating and sleeping needs, and to provide support and guidance.

This study has some limitations. Firstly, in this study, we did not observe any correlation between only sleep problems and social-emotional development. To further validate these findings, more specific questionnaires should be used to evaluate parents’ concerns about eating or sleeping problem. Secondly, parents were the primary reporters in this study, however, for a more comprehensive understanding of children’s lunches and lunch breaks, teachers should be included in the future. Lastly, this study used a convenience sampling method to examine the relationship between parents’ concerns about children’s eating and sleeping problems and children’s social-emotional development. As the current study revealed a gender difference in children’s social-emotional development, which is consistent with previous studies, future research could use the gender of children whose parents have concerns about eating or sleeping problems as a matching variable to examine the relationship between parents’ concerns about eating or sleeping problems and social-emotional development.

## 5 Conclusion

Our research explored the connection between parents’ concerns about eating or sleeping problem and sleeping problems and social-emotional development from the perspective of parenting beliefs. Our findings showed that children whose parents were concerned about only eating or the co-occurrence of eating and sleeping problems was linked to have a higher risk of suspected social-emotional development delay. Therefore, it is essential not only to focus on children’s physical eating and sleeping problems, but also to consider the parenting sense of competence in caring for their child’s eating and sleeping in order to prevent and intervene in children’s social-emotional development.

## Data availability statement

The original contributions presented in the study are included in the article/supplementary material, further inquiries can be directed to the corresponding author.

## Ethics statement

The studies involving humans were approved by the Psychology Ethics Committee of Zhejiang Sci-Tech University. The studies were conducted in accordance with the local legislation and institutional requirements. Written informed consent for participation in this study was provided by the participants' legal guardians/next of kin.

## Author contributions

TH: Conceptualization, Data curation, Investigation, Methodology, Software, Writing – original draft, Writing – review & editing. SL: Funding acquisition, Supervision, Writing – review & editing, Conceptualization, Methodology. JZ: Supervision, Writing – review & editing. LX: Investigation, Writing – review & editing. YZ: Investigation, Writing – review & editing.

## Footnotes

^1^ https://www.renrenedu.cn/survey/index.php?survey_id=27
